# *De novo* assembly of multidrug resistant biofilm forming *Micrococcus luteus* genome from hemodialysis tunneled cuffed catheter tips of patients undergoing renal failure treatment

**DOI:** 10.1128/mra.00095-25

**Published:** 2025-05-30

**Authors:** Rajsekhar Adhikary, Indrani Sarkar, Dhara Patel, Sishir Gang, Uttam Kumar Nath, Saugata Hazra

**Affiliations:** 1 Department of Bioscience and Bioengineering, Indian Institute of Technology-Roorkee 30112, Roorkee, Uttarakhand, India; 2 Department of Medical Oncology and Haematology, AIIMS 442339 , Rishikesh, Uttarakhand, India; 3 Department of Medical Laboratory Technology, Bapubhai Desaibhai Patel Institue of Paramedical Sciences (BDIPS), Charotar University of Science and Technology, CHARUSAT Campus, Anand, Gujarat, India; 4 Department of Nephrology, Muljibhai Patel Urological Hospital 29025, Nadiad, Gujarat, India; 5 Centre for Nanotechnology, Indian Institute of Technology-Roorkee, Roorkee, Uttarakhand, India; Queens College Department of Biology, Queens, New York, USA

**Keywords:** *Micrococcus luteus *HL_Chru_C_3_, whole genome sequencing, antimicrobial resistance, haemodialysis catheter, penicillin-binding proteins

## Abstract

*Micrococcus luteus* HL_Chru_C_3_ was isolated from the hemodialysis tunneled cuffed catheter tip of renal failure patients. Whole-genome sequencing (WGS) revealed a 2,494,573 bp genome with 12 contigs, 72% GC content, and 2,240 protein-coding genes. The computational prediction of penicillin-binding proteins and biofilm-forming signaling gene cassettes may contribute to the resistance mechanisms.

## ANNOUNCEMENT

Renal replacement therapies are lifesaving for renal failure patients. However, bloodstream infections remain the leading cause of death through healthcare-associated infections, with antimicrobial resistance causing 4.95 million deaths globally in 2019 due to the emergence of multidrug-resistant (MDR) pathogens (1–4). The isolate HL_Chru_C_3_ was obtained from the internal lumen of tunneled cuffed hemodialysis catheter tips from renal failure patients at Muljibhai Patel Urological Hospital (ethical approval number MPUH/EC/788/2022) and cultured regularly in nutrient agar medium in aerobic condition. For long-term storage, the stock culture was kept at −20°C in a preservation solution of 20% NB-glycerol. For WGS analysis, the isolate was re-cultured on nutrient agar at 37°C from the previously isolated single colony, and genomic DNA was extracted using the standard phenol-chloroform method before being sent for genomic analysis (5). Identified as *Micrococcus luteus* HL_Chru_C_3_ (GenBank accession number OR682384) through 16S rRNA phylogeny (6) with the 99.52% closest relative was *M. luteus* NCTC 2665 (GenBank accession number CP001628), the isolate exhibited resistance to amikacin, chloramphenicol, ampicillin/sulbactam, piperacillin/tazobactam, doripenem, ertapenem, meropenem, ciprofloxacin, clindamycin, aztreonam, nitrofurantoin, ampicillin, and trimethoprim according to CLSI guidelines (7, Table 1). Due to this multidrug resistance along with the biofilm-forming efficiency of the isolate, WGS analysis was performed using NEBNext Ultra II DNA library prep kit for paired-end library preparation and sequenced on the Illumina NovaSeq 6000 platform (Biokart Genomic Lab, Bengaluru, Karnataka, India), generating 6.2 million reads (2 × 151 bp paired-end format) for HL_Chru_C_3_ (8).

**TABLE 1:** Antibiotic susceptibility profile of *Micrococcus luteus* HL_Chru_C3 against different classes of antibiotics

S. no.	Class	Drug	Mode of action	Resistance profile
1.	Aminoglycosides	Gentamicin	30S subunit Ribosome	Sensitive
2.		Amikacin	30S subunit Ribosome	Resistant
3.		Tobramycin	30S subunit Ribosome	Sensitive
4.	Tetracyclines	Tetracycline	30S subunit Ribosome	Sensitive
5.	Amphenicols	Chloramphenicol	50S subunit Ribosome	Resistant
6.	Lincosamides	Clindamycin	50S subunit Ribosome	Resistant
7.	Fluoroquinolones	Ciprofloxacin	DNA synthesis	Resistant
8.		Levofloxacin	DNA synthesis	Sensitive
9.	Nitrofurantoin	Nitrofurantoin	DNA, RNA, Ribosome	Resistant
10.	Trimethoprim	Trimethoprim	Folic acid synthesis	Resistant
11.	Penicillins	Ampicillin	Cell wall	Resistant
12.	Beta lactam-beta lactamase inhibitor	Ampicillin/sulbactam	Cell wall	Resistant
13.		Piperacillin/tazobactam	Cell wall	Resistant
14.	Second-generation cephalosporins	Cefoxitin	Cell wall	Sensitive
15.	Fourth-generation cephalosporins	Cefepime	Cell wall	Sensitive
16.	Carbapenems	Doripenem	Cell wall	Resistant
17.		Ertapenem	Cell wall	Resistant
18.		Imipenem	Cell wall	Sensitive
19.		Meropenem	Cell wall	Resistant
20.	Monobactams	Aztreonam	Cell wall	Resistant

The adapter removal and trimming of all paired-end reads from the fastq files were executed using TrimGalore v0.6.10, ensuring an average Phred quality score of 38 before assembly (8). The *de novo* assembly was then performed with the Unicycler v0.4.8 assembler (https://github.com/rrwick/Unicycler) (9). Genomic data annotation was carried out using the Prokka v1.14.6 (https://github.com/tseemann/prokka) alongside the NCBI Prokaryotic Genome Annotation Pipeline, producing output files in compliance with standard methods (10). The isolate has a single, 2,494,573 base pairs circular chromosome, with 12 contigs. The genome exhibits high GC content (72%), 2,240 protein-coding sequences, N_50_ count 7, and N_50_ length 139,039 bp. The genome encodes 3 rRNA, 52 tRNA, and 1 tmRNA genes, without plasmid. The antibiotic resistance genes were predicted through the KEGG Automatic Annotation Server for ortholog assignment and pathway mapping using the bi-directional best hit method (11). Default parameters were used for all software unless otherwise specified.

The genome analysis had predicted the presence of carbapenemase, cephalosporin inhibitors (such as *Opp*, *pbp1a/2*, and *FtsI*), and other antibiotic-resistant genes, alongside antibiotic efflux pumps, all contributing to the development of multidrug resistance. Beyond antibiotic resistance, *CRP*, *Vps*, *CyaB*, *Vfr*, *PhnA*, *PhnB*, and *GlgC* proteins were predicted as being involved in signaling for biofilm formation. The *PhnA* and *PhnB* proteins along with *PhzC*, *RpfB*, *Clp*, *Tox*, *Sec*, *OppA*, *Dpp*, *TraC*, *Cyl A*, and *CcfA* were also predicted to be involved in the process of quorum sensing. Further exploration of the genome of this pathogenic, MDR *M. luteus* HL_Chru_C_3_ will enhance our understanding of the antibiotic resistance mechanisms in this bacterium.

## Data Availability

16s rRNA sequence of *M. luteus* HL_Chru_C3 is available under GenBank accession number OR682384. The BioProject and BioSample accession numbers of complete genome of *M. luteus* HL_Chru_C3 are PRJNA1118054 and SAMN41597356 respectively. The NCBI SRA accession number is SRR29262056 and the GenBank accession number JBDZYC000000000.

## References

[1] Wang TZ, Kodiyanplakkal RPL, Calfee DP. 2019. Antimicrobial resistance in nephrology. Nat Rev Nephrol 15:463–481. doi:10.1038/s41581-019-0150-7 31086308 PMC7269065

[2] Murray CJL, Ikuta KS, Sharara F, Swetschinski L, Robles Aguilar G, Gray A, Han C, Bisignano C, Rao P, Wool E, et al.. 2022. Global burden of bacterial antimicrobial resistance in 2019: a systematic analysis. The Lancet 399:629–655. doi:10.1016/S0140-6736(21)02724-0 PMC8841637 35065702

[3] Caini S, Hajdu A, Kurcz A, Borocz K. 2013. Hospital-acquired infections due to multidrug-resistant organisms in Hungary, 2005-2010. Euro Surveill 18:20352. 23324427

[4] Cornejo-Juárez P, Vilar-Compte D, Pérez-Jiménez C, Ñamendys-Silva SA, Sandoval-Hernández S, Volkow-Fernández P. 2015. The impact of hospital-acquired infections with multidrug-resistant bacteria in an oncology intensive care unit. Int J Infect Dis 31:31–34. doi:10.1016/j.ijid.2014.12.022 25528484

[5] Sambrook J, Russell D. 2001. Molecular cloning: a laboratory manual. 2nd ed. Cold Spring Harbor Laboratory, Cold Spring Harbor.

[6] Bandyopadhyay B, Das S, Mitra PK, Kundu A, Mandal V, Adhikary R, Mandal V, Mandal NC. 2022. Characterization of two new strains of Lactococcus lactis for their probiotic efficacy over commercial synbiotics consortia. Braz J Microbiol 53:903–920. doi:10.1007/s42770-022-00685-6 35138631 PMC9151986

[7] Clinical and Laboratory Standard Institute (CLSI). 2020. M100:performance standards for anti-microbial susceptibility testing. 30th ed

[8] Pandey NK, Hazra S. 2024. Complete genome sequence of carbapenem-resistant pathogenic Klebsiella aerogenes strain CH7 isolated from vermicompost. Microbiol Resour Announc 13:e0128423. doi:10.1128/mra.01284-23 38700350 PMC11237382

[9] Wick RR, Judd LM, Gorrie CL, Holt KE. 2017. Unicycler: resolving bacterial genome assemblies from short and long sequencing reads. PLoS Comput Biol 13:e1005595. doi:10.1371/journal.pcbi.1005595 28594827 PMC5481147

[10] Tatusova T, DiCuccio M, Badretdin A, Chetvernin V, Nawrocki EP, Zaslavsky L, Lomsadze A, Pruitt KD, Borodovsky M, Ostell J. 2016. NCBI prokaryotic genome annotation pipeline. Nucleic Acids Res 44:6614–6624. doi:10.1093/nar/gkw569 27342282 PMC5001611

[11] Moriya Y, Itoh M, Okuda S, Yoshizawa AC, Kanehisa M. 2007. KAAS: an automatic genome annotation and pathway reconstruction server. Nucleic Acids Res 35:W182–5. doi:10.1093/nar/gkm321 17526522 PMC1933193

